# Moderate-to-high risk of obstructive sleep apnea with excessive daytime sleepiness is associated with postoperative neurocognitive disorders: a prospective one-year follow-up cohort study

**DOI:** 10.3389/fnins.2023.1161279

**Published:** 2023-05-31

**Authors:** Wenwen Wu, Lihui Pu, Xiuying Hu, Qian Chen, Guan Wang, Yanyan Wang

**Affiliations:** ^1^West China School of Nursing, Sichuan University, Chengdu, Sichuan, China; ^2^Innovation Center of Nursing Research, Nursing Key Laboratory of Sichuan Province, West China Hospital, Sichuan University, Chengdu, Sichuan, China; ^3^Menzies Health Institute Queensland & School of Nursing and Midwifery, Griffith University, Brisbane, QL, Australia; ^4^Department of Geriatrics and National Clinical Research Center for Geriatrics, West China School of Nursing, West China Hospital, Sichuan University, Chengdu, Sichuan, China; ^5^Science and Technology Department, National Clinical Research Center for Geriatrics, West China Hospital, Sichuan University, Chengdu, Sichuan, China

**Keywords:** obstructive sleep apnea, excessive daytime sleepiness, perioperative neurocognitive disorders, older adults, surgery

## Abstract

**Background:**

Few studies found that obstructive sleep apnea (OSA) may be related to postoperative neurocognitive disorders (PND) including postoperative delirium (POD) and cognitive decline (POCD) in the early postoperative period. However, the results are controversial and need further verification, and no research has explored the effect of OSA on the incidence of PND during the 1-year follow-up periods. Furthermore, OSA patients with excessive daytime sleepiness (EDS) as a severe phenotype have more significant neurocognitive impairments, but the relationship between OSA with EDS and PND within 1 year after surgery has not been studied.

**Objectives:**

To explore the effect of moderate-to-high risk of OSA and the moderate-to-high risk of OSA with EDS on PND within 1 year after surgery.

**Methods:**

In this prospective cohort study, including 227 older patients, moderate-to-high risk of OSA (using STOP-BANG), subjective EDS (using Epworth Sleepiness Scale), and objective EDS (using Actigraphy) were selected as exposures. Key outcomes included POD during hospitalization (using Confusion Assessment Method-Severity), POCD at discharge, 1-month and 1-year after surgery (using Mini-Mental State Examination and Telephone Interview for Cognitive Status-40). We applied multiple logistic regression models to estimate the effect of moderate-to-high risk of OSA and moderate-to-high risk of OSA with EDS on PND.

**Results:**

In the multivariate analysis, moderate-to-high risk of OSA was not associated with POD during hospitalization and POCD at discharge, 1-month, and 1-year after surgery (*p* > 0.05). However, the moderate-to-high risk of OSA with subjective EDS was related to POCD at discharge compared to the moderate-to-high risk of OSA or normal group (no moderate-to-high risk of OSA and no EDS) (*p* < 0.05). In addition, moderate-to-high risk of OSA with objective EDS was associated with POCD at discharge, 1-month, and 1-year postoperatively compared to the moderate-to-high risk of OSA or normal group (*p* < 0.05).

**Conclusion:**

Moderate-to-high risk of OSA with EDS, not moderate-to-high risk of OSA alone, was a clinically helpful predictor for POCD within 1-year after surgery and should be routinely assessed before surgery.

## Introduction

Obstructive sleep apnea (OSA), characterized by chronic intermittent hypoxemia and sleep fragmentation, is primarily caused by episodes of complete or partial airway obstruction during sleep (Malhotra and White, 2002) OSA occurs in 10%–20% of the population, increases with age, and peaks after 60 (Bixler et al., 1998, 2001; Peppard et al., 2013). Excessive daytime sleepiness (EDS) is the most common symptom in patients with OSA, and its clinical features are daytime drowsiness, reduced wakefulness, and decreased vigilance (Sauter et al., 2000). EDS has a prevalence of 16%–22% in OSA patients and often means patients at high risk of OSA. Previous studies showed that OSA patients with EDS as a distinct clinical phenotype had lower oxygen saturation and more sleep disturbance than those without EDS (Zhou et al., 2016). Many studies recommended Epworth Sleepiness Scale (ESS) for measuring subjective EDS and multiple sleep latency test (MSLT) for objective EDS, respectively (Ren et al., 2016). Daytime naps recorded by actigraphy also reflect objective EDS, (Leng et al., 2018) which is a compensatory reaction to nocturnal sleep fragmentation, and it can also be a marker of EDS in older adults (Hsieh et al., 2016).

Previous studies have shown that OSA is associated with impaired cognitive function, including attention, alertness, memory, and executive function (Andrade et al., 2018; Stepnowsky et al., 2019; Bubu et al., 2020; Léger and Stepnowsky, 2020; Liu et al., 2020). EDS may also contribute to cognitive impairment in OSA patients, especially attention and memory function because EDS is strictly related to sleep deprivation and fragmentation (Steiropoulos et al., 2019). Many studies reported that OSA patients with EDS have more significant cognitive impairments involving attention and vigilance, learning and memory, and executive function than those without EDS (Zhou et al., 2016). Several pathophysiological factors, such as intermittent hypoxia, systemic inflammation, and oxidative stress, may influence cognitive function in OSA patients. It has been speculated that the interaction between EDS and hypoxemia contributes to neurocognitive deficits (Roche, 2016).

The prevalence of OSA in the surgical population is higher than in the normal population and varies widely among different surgery patients (Van Onselen et al., 2013; Zaremba et al., 2016). Multiple perioperative factors such as anesthetics and analgesics can worsen OSA. In addition, reorganization of sleep architecture with rapid eye movement sleep rebound attributed to postoperative disrupted, reduced, and poor-quality sleep leads to exacerbation of OSA and EDS (Finkel et al., 2009; Young et al., 2009). Add to them downstream reactions of surgery stress and anesthetics such as inflammatory responses, oxidative stress, metabolic disorders, and neuronal damage, the postoperative complications are more common in surgical populations with OSA than those without OSA (Memtsoudis et al., 2011; Roggenbach et al., 2014; Lam et al., 2017; Chan et al., 2019).

In recent years, OSA as a substantial risk factor for postoperative delirium and cognitive decline has aroused scholars’ attention. Postoperative neurocognitive disorder (PND) is a term that refers to cognitive impairment associated with anesthesia and surgery, including the acute event (postoperative delirium, POD) and cognitive decline diagnosed up to 1 year after surgery (postoperative cognitive dysfunction, POCD) (Evered et al., 2018). POD occurs in 13%–34% of surgical patients, and POCD occurs in 7%–50%, depending on surgery type and surgical populations (Postler et al., 2011; Norkienė et al., 2013; Krenk et al., 2014; Aceto et al., 2015; Goettel et al., 2017; Kotfis et al., 2018; Wang C. G. et al., 2018; Chaiwat et al., 2019; Guo et al., 2020; Kang et al., 2020; Wang et al., 2020; Yang et al., 2020; Li et al., 2021). Previous studies showed that the prevalence of POD and POCD were 14% and 19% among elderly patients undergoing gastrointestinal surgery, respectively (Yang et al., 2020; Li et al., 2021). Enough evidence has shown that the development of PND is associated with inflammation, oxidative stress, metabolic disorders, increased blood–brain barrier permeability, and neuronal damage (Hudetz et al., 2011; Subramaniyan and Terrando, 2019; Lin et al., 2020). Patients who developed PND have an increased risk of postoperative complications and a worse prognosis (Abelha et al., 2013; Abawi et al., 2016; Lingehall et al., 2017; Ruggiero et al., 2017; Kotfis et al., 2018; Racine et al., 2018; Shi et al., 2019; Boone et al., 2020; Cai et al., 2020).

So far, 10 studies have explored the correlation between OSA and POD. Four studies confirmed that OSA increased the risk of POD (Flink et al., 2012; Roggenbach et al., 2014; Nadler et al., 2017; Wu et al., 2022) while the remaining six did not identify their association (Gupta et al., 2001; Wang S. et al., 2018; Strutz et al., 2019; Tafelmeier et al., 2019; King et al., 2020; Oldham et al., 2021). Only three human studies and three animal experiments have explored the correlation between OSA and POCD. Two human studies showed that the incidence of POCD before discharge in OSA patients was lower than that of non-OSA patients (Wagner et al., 2018, 2021) and one human study showed that OSA patients had a higher risk of POCD in the early postoperative period than patients without OSA (Wu et al., 2022). Three animal models also suggested that the incidence of POCD was higher in OSA mice than that in non-OSA mice (Dong et al., 2018; Zhang et al., 2019; Mei et al., 2020). However, previous studies assessed cognitive function within 2 days after extubating with a lack of long-term postoperative follow-up cognitive assessments at 1-month or 1-year after surgery. In addition, no study, to our knowledge, has shown the effect of moderate-to-high risk of OSA combined with EDS on PND within 1 year after surgery. Therefore, the first aim of our study was to explore the impact of moderate-to-high risk of OSA on the PND within 1 year postoperatively, and the second aim was to investigate the effect of moderate-to-high risk of OSA with EDS on PND within 1 year after surgery. We hypothesized that moderate-to-high risk of OSA with EDS was associated with the development of PND in older adults undergoing gastrointestinal surgery.

## Materials and methods

### Study design

With the ethical approval from the institutional review board of West China Hospital, this single-center prospective cohort study was conducted from June 4, 2019, to October 14, 2021, at a gastrointestinal surgical unit (≥80 beds, approximately 35 nurses, and 20 surgeons) of West China Hospital in Chengdu, China. All participants or their legal representatives provided written informed consent before the study. Patients did not receive financial compensation. Study methods and results are reported following the Strengthening the Reporting of Observational Studies in Epidemiology (STROBE) Statement for prospective cohort studies (Vandenbroucke et al., 2007).

### Participants

**Figure 1:**
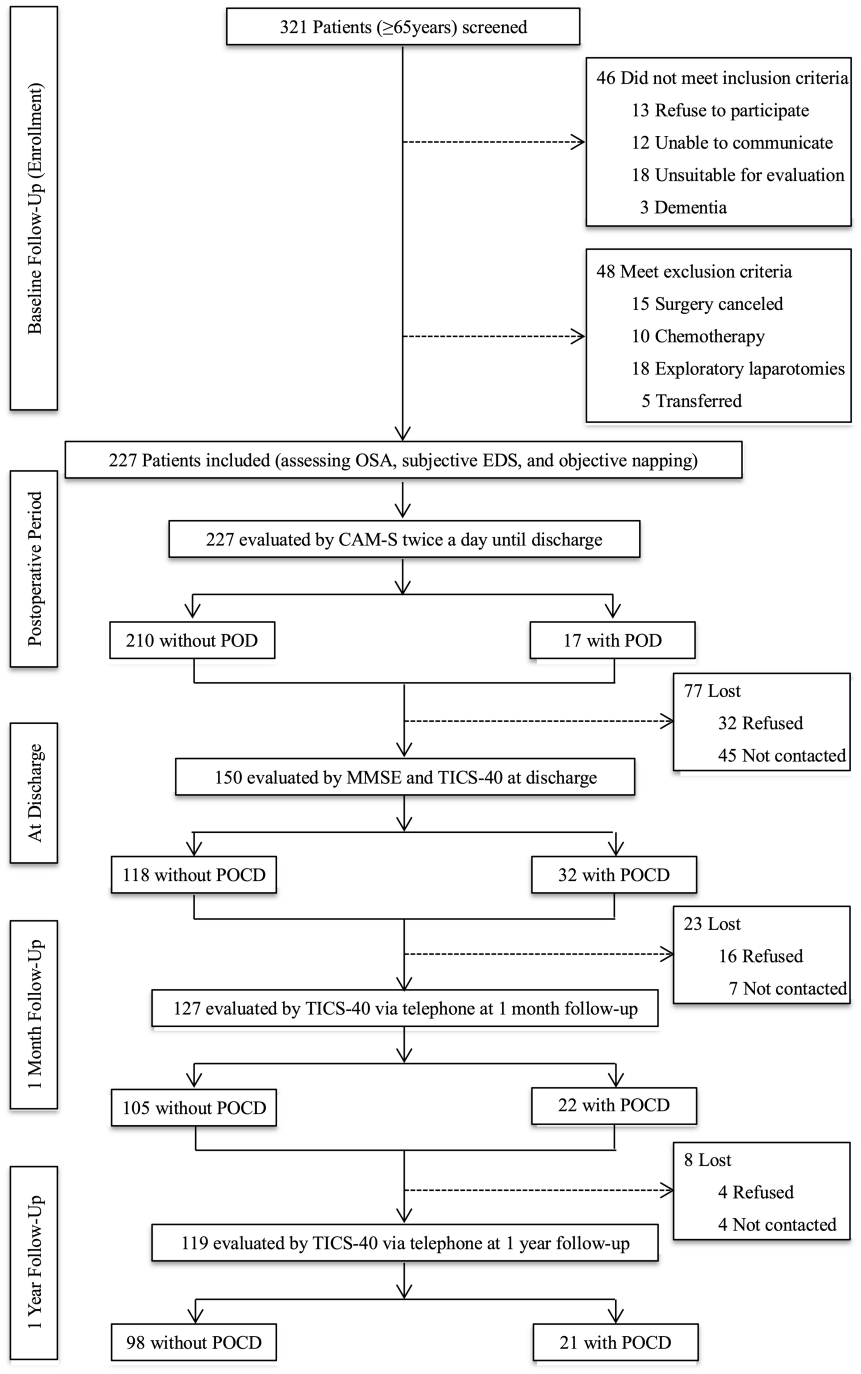
Study flow diagram. OSA, obstructive sleep apnea; EDS, excessive daytime sleepiness; CAM-S, Confusion Assessment Method-Severity; POD, postoperative delirium; MMSE, Mini-Mental State Examination; TICS-40, Telephone Interview for Cognitive Status-40; POCD, postoperative cognitive dysfunction.

We screened older adults (≥65 years) who were scheduled for stomach and intestinal surgery between June 4, 2019, and October 14, 2020. Patients who finally underwent the gastrointestinal procedure with at least a 3-day hospital stay, without communication barriers, and had a suitable condition for evaluation were included. The exclusion criteria are as follows: (1) cognitive impairment with a Mini-Mental State Examination (MMSE) scores less than 20; (2) delirium (assessed by Confusion Assessment Method-Severity (CAM-S Short Form) at baseline; (3) a documented history of severe psychosis such as severe depression, severe anxiety, autism, and schizophrenia; (4) a terminal condition with a life expectancy of fewer than 6 months (metastatic cancer, multiple organ failure, or receiving chemotherapy); (5) exploratory laparotomy without tumor removal or transferring to other departments after the operation; and (6) alcohol abuse or dependence within the last 3 months. Patients were also excluded if they had missing baseline, procedural, or outcome data (Figure 1).

### Assessments

The face-to-face evaluation was performed at the ward of the gastrointestinal surgery department on admission day by a researcher who received training in psychiatry and assessment methods. The baseline assessment included risks of OSA, subjective EDS, and objective napping. Then, the researcher and trained nurses assessed POD using the CAM-S twice daily (between 2–6 p.m. and 2–8 a.m.) until postoperative day seven or discharge. Another trained researcher blinded to the POD results performed postoperative cognitive assessments at discharge using MMSE and Telephone Interview for Cognitive Status-40 (TICS-40). Postoperative cognitive function at one month and one year after surgery were also evaluated using TICS-40 by telephone. The criteria for loss to follow-up were as follows: (1) refused to participate; (2) cannot be contacted; and (3) died (Figure 1).

### Exposure

The STOP-BANG questionnaire has good sensitivity and high diagnostic accuracy for detecting OSA in Asian populations (Chan et al., 2019). A STOP-BANG score of 3–8 indicates a moderate-to-high risk of OSA (OSA group) among the Chinese people, and a 0–2 score means a low risk of OSA (non-OSA group) (Chan et al., 2019). Subjective EDS was defined as an ESS score ≥ 11, and the Chinese version of ESS was a reliable and valid tool for measuring subjective EDS (Pak et al., 2017). Objective napping was measured using an Actiwatch-2 (Phillips Respironics Mini-Mitter), worn continuously on the non-dominant wrist for a minimum of two consecutive 24 h periods before operation. Participants also completed sleep logs for the period they wore the actigraphy and reported information regarding times they napped. Data for devices were collected in 1-min epochs and scored using Actiware software version 6.0.9 (Phillips Respironics, Bend, OR). Daily napping duration was calculated by summing up the time of napping periods throughout the day and averaging across all recording days. A nap ≥1 h/day during the day was considered objective napping (Leng et al., 2018). For convenience, subjective EDS and objective napping will be referred to as “EDS” throughout the rest of the manuscript.

### Cognitive function

The CAM-S provides a new delirium severity measure with strong psychometric properties and associations with important clinical outcomes. The Short Form includes the same four features as CAM: acute change or fluctuation, inattention, disorganized thinking, and altered level of consciousness. The diagnostic criteria of delirium are that the first and second criteria must be present, coming with the third or fourth criteria. The sum score of CAM-S Short Form ranges from 0 (no) to 7 (most severe) (Inouye et al., 2014). The Chinese version of the CAM-S demonstrated good reliability and validity in evaluating postoperative delirium among hospitalized Chinese geriatric patients (Mei et al., 2019). MMSE is scored from 0 to 30, with a test–retest reliability of 0.8–0.1 and a scorer reliability of 0.9–1.0. The Chinese-language version of the MMSE has been used in elderly Chinese, showing a high sensitivity of 87.6% and specificity of 80.8% (Li et al., 2016). MMSE is the instrument most widely used in screening for cognitive problems in hospitalized patients (Borchers et al., 2021). The Chinese version of the TICS-40 was previously validated and scored on a scale of 0 to 40, with a higher score indicating better cognitive function (Liu et al., 2021). A TICS-40 score ≤ 20 was defined as mild cognitive impairment, and a score ≤ 12 referred to dementia, according to a previous study (Wang et al., 2021). To control for learning effects (improvements over time with repeated testing), we applied an accepted approach that is using alternate forms of MMSE and TICS-40 (Munjir et al., 2015; Lee et al., 2022). With the structures and scores unchanged, we changed the contents of questions at every postoperative cognition evaluation according to previous studies (e.g., “Please raise your hands,” replaced the phrase, “Please close your eyes”) (Katzman et al., 1988; Fong et al., 2009).

### Confounding factors

We also collected information on demographic characteristics, including age, sex, educational level, body mass index (BMI), smoking, drinking, exercise behavior, hypertension, and the American Society of Anesthesiologists Physical Status Classification System (ASA). The Frail Scale (FS) has an excellent test–retest reliability of 0.7 in the Chinese community (Yuan et al., 2021). The total score of 5 items is 0 means health status, 1–2 means pre-frail, and 3–5 means frail (Yuan et al., 2021). Mini Nutritional Assessment-Short Form (MNA-SF) is a standard method to evaluate the nutritional status of the elderly. The Chinese version of MNA-SF performed well, and a score of 12–14 points is normal nutritional status, 8–11 points are at risk of malnutrition, and 0–7 points indicate malnutrition (Amasene et al., 2021; Yao et al., 2022). Furthermore, intraoperative data (operation time, anesthetic time, and general anesthesia method) (intravenous, inhalation, or a combination of both ways) were accessed from medical records.

### Outcomes

The primary outcomes were the incidence of PND, including POD during hospitalization, POCD at discharge, 1 month, and 1 year after surgery. Delirium appearing on one of all postoperative days before discharge was regarded as POD (Saczynski et al., 2014; Schmitt et al., 2015). The term POCD described in the literature ranged from within 24 h after surgery to 12 months (Evered and Silbert, 2018). In general, 1-month has been taken as a time in which the acute effect of surgery and anesthesia has abated (Evered and Silbert, 2018). POCD was mainly developed early after surgery and fully recovered cognitive function 3 months after surgery (Evered et al., 2018; Evered and Silbert, 2018). Few patients had cognitive decline persisting up to 1 year after surgery; this may indicate a possible progression to dementia (Evered et al., 2018). Therefore, early POCD was assessed at discharge and defined as at least one cognitive performance score (MMSE and the TICS-40 test) declined 2 points or more compared to the baseline score following previously used standards (Hollinger et al., 2021; Suraarunsumrit et al., 2022). POCD at 1 month and 1 year were defined as a decline in 1-month and 1-year TICS-40 performance of two points or more compared to preoperative TICS-40 score, respectively (Hollinger et al., 2021; Suraarunsumrit et al., 2022; van Zuylen et al., 2023).

### Statistical analysis

For univariate analysis, descriptive data were summarized using proportions for categorical data and means with standard deviation (SD) or medians with interquartile range (IQR) for continuous data. Comparison between PND and non-PND groups using *χ*^2^ analysis or Fisher exact probability test for categorical variables and *t*-test or, if the variables were not normally distributed, the Mann–Whitney test for continuous variables. The Wilcoxon rank sum tests were also used for ranked data. For multivariable analysis, we applied a multiple logistic regression model to adjust potential modifiers and estimate the effect of moderate-to-high risk of OSA, subjective EDS, and objective napping for PND. Furthermore, to examine the joint effect of moderate-to-high risk of OSA and EDS, we performed logistic regression models that included five dummy variables to represent all six possible combinations of moderate-to-high risk of OSA and EDS. We first used the moderate-to-high risk of OSA as a reference group, then we used no moderate-to-high risk of OSA with no subjective EDS and no objective napping as a reference group. All participants in our study used combined intravenous inhalational anesthesia, so the anesthetic method was not included in the regression model. Finally, confounding factors included age, BMI, FS score, MNA-SF score, and preoperative MMSE score in model 1; and confounding factors included age, BMI, FS score, MNA-SF score, preoperative MMSE, sex, and educational level in model 2. Statistical analysis and data visualization were performed using IBM SPSS, version 25 (IBM Corp, Armonk, NY, United States) and GraphPad Prism v.9 (GraphPad Software, San Diego, CA, United States). All tests were two-sided, and a *p* value < 0.05 was designated as statistically significant.

## Results

### Characteristics of the study population based on PND categories

The descriptive statistics for the participants’ perioperative characteristics according to the presence of PND are summarized in Table 1. A total of 227 participants (67.4% male; median (IQR) age: 69 (66–73) years) were included in the POD analyses, and 17 patients developed POD. Of 150 older adults (70.0% male; median (IQR) age: 69 (66–73) years) analyzed for POCD at discharge, 32 people had POCD. We finally included 127 (68.6% male; median (IQR) age: 69 (66–73) years) and 119 (63.9% male; median (IQR) age: 70 (67–75) years) patients for POCD at 1 month and 1 year analysis, respectively. The number of patients who developed POCD at 1 month was 22, and the cases of patients who existed POCD at 1 year were 21. The postoperative cognitive assessment results are shown in Supplementary Table S1. There was no significant difference among the PND (POD, POCD at discharge, POCD at 1 month, and POCD at 1 year) and non-PND groups in preoperative characteristic and surgical data except for the MNA-SF scores and MMSE scores. The MNA-SF scores were higher in the POD group than in the non-POD group. In addition, MMSE scores were lower in the POCD group compared to the non-POCD group at 1 year.

**Table 1:** Baseline characteristics of the study population by postoperative neurocognitive disorders categories.

Characteristics	POD (*N* = 227)				POCD at discharge (*N* = 150)				POCD at 1 month (*N* = 127)				POCD at 1 year (*N* = 119)			
	Total (*N* = 227)	No-POD (*n* = 210)	POD (*n* = 17)	*P*-value	Total (*N* = 150)	No-POCD (*n* = 118)	POCD (*n* = 32)	*P*-value	Total (*N* = 127)	No-POCD (*n* = 105)	POCD (*n* = 22)	*P*-value	Total (*N* = 119)	No-POCD (*n* = 98)	POCD (*n* = 21)	*P*-value
Age, year	69 (66–73)	70 (67–73)	69 (66–73)	0.634	69 (66–73)	68 (66–72)	70 (66–73)	0.466	69 (66–73)	69 (66–72)	70 (66–73)	0.682	70 (67–75)	69 (66–71)	70 (67–75)	0.357
Male	153 (67.4)	141 (67.1)	12 (70.6)	0.771	105 (70.0)	84 (71.2)	21 (65.6)	0.543	85 (66.5)	72 (68.6)	13 (59.1)	0.390	76 (63.9)	63 (64.3)	13 (61.9)	0.837
Educational level^a^				0.506				0.850				0.144				0.740
Primary	90 (39.6)	83 (39.5)	7 (41.2)		62 (41.3)	50 (42.4)	12 (37.5)		49 (38.6)	37 (35.2)	12 (54.5)		44 (37.0)	35 (35.7)	9 (42.9)	
Secondary	106 (46.7)	101 (48.1)	5 (29.4)		65 (43.3)	49 (41.5)	16 (50.0)		57 (44.9)	50 (47.6)	7 (31.8)		57 (47.9)	51 (52.0)	6 (28.6)	
Higher	31 (13.7)	26 (12.4)	5 (29.4)		23 (15.3)	19 (16.1)	4 (12.5)		21 (16.5)	18 (17.1)	3 (13.6)		18 (15.1)	12 (12.2)	6 (28.6)	
BMI	22.7 ± 2.9	22.6 ± 3.0	23.4 ± 2.5	0.269	22.7 ± 3.1	22.6 ± 3.2	22.9 ± 2.7	0.639	22.6 ± 3.1	22.6 ± 3.2	22.6 ± 2.6	0.943	22.5 ± 2.8	22.3 ± 2.8	23.5 ± 2.5	0.087
Smoke	80 (35.2)	76 (36.2)	4 (23.5)	0.293	57 (38.0)	47 (39.8)	10 (31.3)	0.375	43 (33.9)	37 (35.2)	6 (27.3)	0.473	35 (29.4)	29 (29.6)	6 (28.6)	0.926
Drink	57 (25.1)	53 (25.2)	4 (23.5)	0.876	42 (28.0)	35 (29.7)	7 (21.9)	0.384	31 (24.4)	26 (24.8)	5 (22.7)	0.840	25 (21.0)	22 (22.4)	3 (14.3)	0.405
Exercise				0.241				0.855				0.242				0.976
No	32 (14.1)	28 (13.3)	4 (23.5)		23 (15.3)	17 (14.4)	6 (18.8)		18 (14.2)	12 (11.4)	6 (27.3)		17 (14.3)	14 (14.3)	3 (14.3)	
Occasionally	11 (4.8)	10 (4.8)	1 (5.9)		9 (6.0)	8 (6.8)	1 (3.1)		7 (5.5)	7 (6.7)	0 (0.0)		6 (5.0)	5 (5.1)	1 (4.8)	
Every day	184 (81.1)	172 (81.9)	12 (70.6)		118 (78.7)	93 (78.8)	25 (78.1)		102 (80.3)	86 (81.9)	16 (72.7)		96 (80.7)	79 (80.6)	17 (81.0)	
Hypertension	116 (51.1)	109 (51.9)	7 (41.2)	0.395	93 (62.0)	71 (60.2)	22 (68.8)	0.375	76 (59.8)	62 (59.0)	14 (63.6)	0.690	84 (70.6)	70 (71.4)	14 (66.7)	0.664
ASA status				0.204				0.448				0.692				0.432
I-II	127 (55.9)	115 (54.8)	12 (70.6)		88 (58.7)	67 (56.8)	21 (65.6)		78 (61.4)	65 (61.9)	13 (59.1)		68 (57.1)	54 (55.1)	14 (66.7)	
III	99 (43.6)	94 (44.8)	5 (29.4)		61 (40.7)	51 (43.2)	10 (31.3)		48 (37.8)	40 (38.1)	8 (36.4)		50 (42.0)	44 (44.9)	6 (28.6)	
IV	1 (0.4)	1 (0.5)	0 (0.0)		1 (0.7)	0 (0.0)	1 (3.1)		1 (0.8)	0 (0.0)	1 (4.5)		1 (0.8)	0 (0)	1 (4.8)	
FS score	0 (0–1)	0 (0–1)	0 (0–1)	0.824	0 (0–1)	0 (0–1)	0 (0–1)	0.306	0 (0–1)	0 (0–1)	0 (0–1)	0.370	0 (0–1)	0 (0–1)	0 (0–1)	0.574
MNA-SF scores	12 (10–13)	13 (11–14)	11 (10–13)	**0.007**	12 (10–13)	12 (10–13)	12 (10–13)	0.893	12 (10–13)	12 (10–13)	12 (10–13)	0.938	12 (10–13)	12 (10–13)	12 (10–13)	0.169
MMSE scores	29 (27–30)	29 (27–30)	28 (27–29)	0.542	29 (27–30)	29 (27–30)	29 (27–29)	0.181	29 (27–30)	29 (26–30)	29 (28–30)	0.454	29 (27–30)	29 (28–30)	28 (27–29)	**0.017**
TICS-40 scores	33 (32–34)	33 (32–34)	33 (32–34)	0.214	33 (32–34)	33 (32–34)	33 (32–34)	0.735	33 (32–34)	33 (32–34)	33 (31–34)	0.671	33 (31–34)	33 (31–34)	33 (32–34)	0.297
Operation times, h^b^	2.8 ± 1.1	2.7 ± 1.1	3.2 ± 1.0	0.117	2.8 ± 1.1	2.8 ± 1.1	2.7 ± 1.2	0.566	2.7 ± 1.0	2.7 ± 1.0	2.8 ± 1.0	0.657	2.7 ± 1.1	2.8 ± 1.1	2.5 ± 1.1	0.313
Anesthetic time, h^c^	4.0 ± 1.3	4.0 ± 1.3	4.2 ± 1.0	0.617	4.1 ± 1.3	4.1 ± 1.2	4.2 ± 1.6	0.706	4.0 ± 1.2	4.0 ± 1.2	4.2 ± 1.3	0.460	4.0 ± 1.3	4.0 ± 1.2	4.0 ± 1.5	0.787

The normally distributed variables are presented as mean ± SD, non-normally distributed variables are presented as median (IQR), the rank variables and categories variables are presented as frequency (%). The normally distributed data were compared using unpaired 2-tailed *t*-test, the non-normally distributed data and rank data were compared with Mann–Whitney U test, and the categories data were compared using the Pearson chi-square test. The bold values indicated that *p* value <0.05.

a Primary educational level included an illiterate or primary school diploma; secondary educational level consisted of a junior high school, senior high school, or technical secondary school diploma; high educational level meant having a college, bachelor, master’s degree or above.

b Operation time started from skin discission to incision close except closed reduction.

c Anesthetic time started from the injection of anesthetic until the patient woke up.

POD, postoperative delirium; POCD, postoperative cognitive dysfunction; BMI, body mass index; ASA, American Society of Anesthesiologists; FS, frail scale; MNA-SF, mini nutritional assessment-short form; MMSE, Mini-Mental State Examination; TICS-40, Telephone Interview for Cognitive Status-40; SD, standard deviation; IQR, interquartile range.

### Association between the moderate-to-high risk of OSA, subjective EDS, and objective napping

Correlations between the moderate-to-high risk of OSA, subjective EDS, and objective napping are shown in Table 2. The results (Table 2A) showed that subjective EDS was significantly associated with moderate-to-high risk of OSA and objective napping. However, as shown in Table 2B, patients with objective napping had a higher risk of OSA without statistical significance.

**Table 2:** (A) Association between the moderate-to-high risk of OSA, subjective EDS, and objective napping. (B) Association between the moderate-to-high risk of OSA and objective napping.

A												
OSA	POD (*N* = 227)			POCD at discharge (*N* = 150)			POCD at 1 month (*N* = 127)			POCD at 1 year (*N* = 119)		
	No-EDS (*n* = 179)	EDS ^c^ (*n* = 48)	*P*-value	No-EDS (*n* = 118)	EDS (*n* = 32)	*P*-value	No-EDS (*n* = 99)	EDS (*n* = 28)	*P*-value	No-EDS (*n* = 94)	EDS (*n* = 25)	*P*-value
Moderate-to-high risk of OSA^a^	116 (64.8)	47 (97.9)	**<0.001**	84 (71.2)	31 (96.9)	**0.002**	67 (67.7)	27 (96.4)	**0.002**	56 (59.6)	25 (100)	**<0.001**
Objective napping^b^	68 (38.0)	27 (56.3)	**0.023**	42 (35.6)	19 (59.4)	**0.015**	33 (33.3)	16 (57.1)	**0.022**	33 (35.1)	12 (48.0)	0.237

B												
OSA	POD (*N* = 227)			POCD at discharge (*N* = 150)			POCD at 1 month (*N* = 127)			POCD at 1 year (*N* = 119)		
	No-naps (*n* = 132)	Naps (*n* = 95)	*P*-value	No-naps (*n* = 89)	Naps (*n* = 61)	*P*-value	No-naps (*n* = 78)	Naps (*n* = 49)	*P*-value	No-naps (*n* = 74)	Naps (*n* = 45)	*P*-value
Moderate-to-high risk of OSA	94 (71.2)	69 (72.6)	0.815	67 (75.3)	48 (78.7)	0.628	57 (73.1)	37 (75.5)	0.761	50 (67.6)	31 (68.9)	0.881

The categories variables were presented as frequency (%) and compared using the Pearson chi-square test, or when at least one of the cells contingency tables had an expected *n* < 5, the Fisher exact probability test. The rank data were compared with the Mann–Whitney U test. The bold values indicated that *p* value <0.05.OSA, obstructive sleep apnea; EDS, excessive daytime sleepiness; POD, postoperative delirium; POCD, postoperative cognitive dysfunction; ESS, Epworth sleepiness scale.

a Moderate-to-high risk of OSA defined as a STOP-BANG score of 3–8.

b Objective napping is considered as sleep time of more than 60 min between 9 a.m. and 7 p.m.

c EDS meant subjective EDS and was defined as an ESS score > 10.

### Effect of the moderate-to-high risk of OSA, subjective EDS, or objective napping on PND

**Figure 2:**
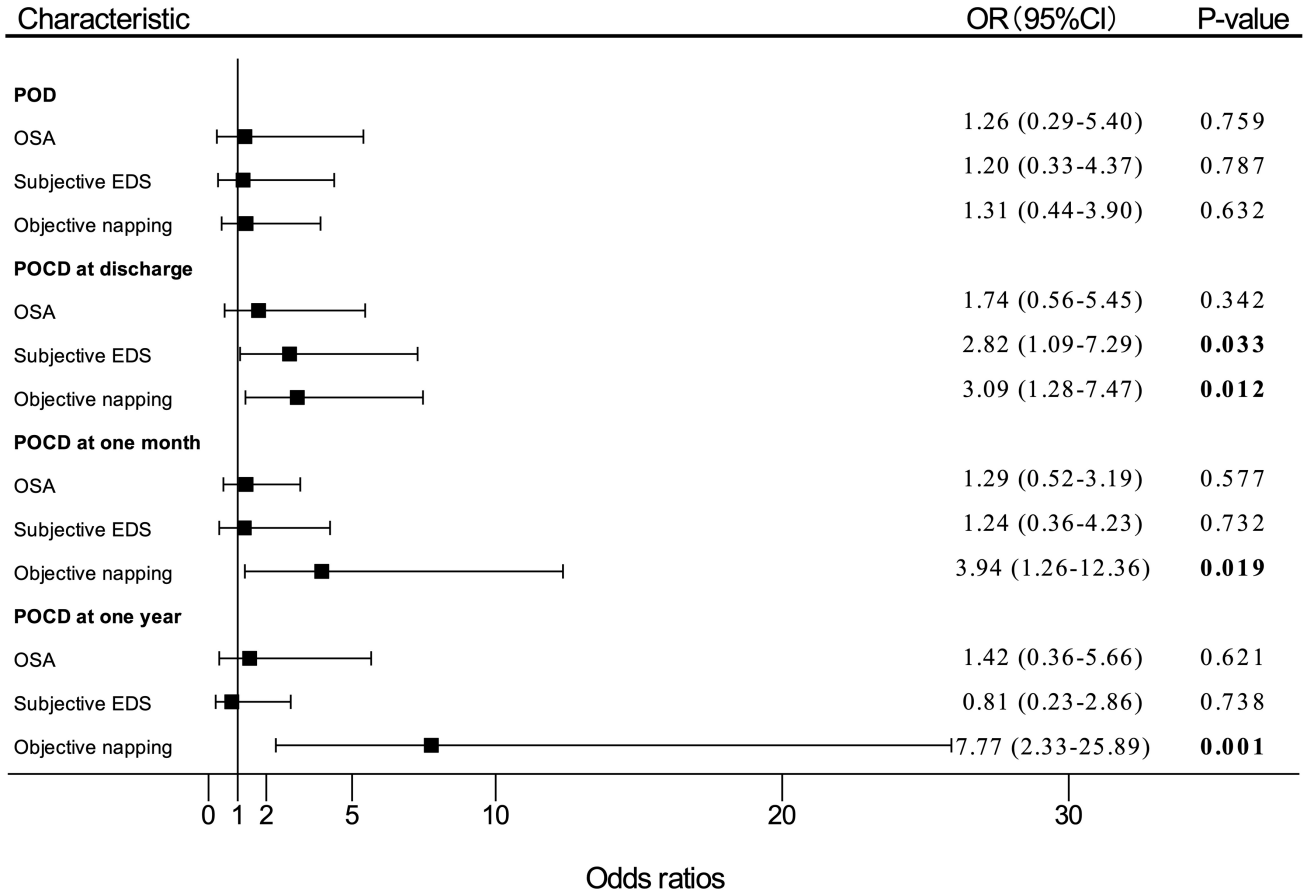
Adjusted (OR) and 95%CI of the effect of OSA or EDS on PND. Estimates were obtained by logistic regression, after adjusting for the significant terms: age, BMI, FS score, MNA-SF score, and MMSE score. “OSA” means patients with moderate-to-high risk of OSA. OR, odds ratio; OSA, obstructive sleep apnea; EDS, excessive daytime sleepiness; PND, perioperative neurocognitive disorders; BMI, body mass index; FS, frail scale; MNA-SF, mini nutritional assessment-short form; MMSE, Mini-Mental State Examination; POD, postoperative delirium; POCD, postoperative cognitive dysfunction.

Univariate analysis was performed, and the results are shown in Supplementary Table S2. Multivariate analysis results in model 1 are shown in Figure 2, and multivariate analysis results in model 2 are shown in Supplementary Figure S1.

In multivariate logistic regression analysis for PND, patients who reported subjective EDS had nearly three times (OR, 2.82; 95% CI, 1.09–7.29; *p* = 0.033) as likely to develop POCD at discharge compared with patients without subjective EDS. The participants who had objective napping also had almost three times (OR, 3.09; 95% CI, 1.28–7.47; *p* = 0.012) as likely to develop POCD at discharge compared with those without objective napping. In addition, the incidence of POCD at 1 month after surgery significantly increased by 294% (OR, 3.94; 95% CI, 1.26–12.36; *p* = 0.019) among patients with objective napping than those without napping. Objective napping also caused a 677% (OR, 7.77; 95%CI, 2.33–25.89; *p* = 0.001) increase in the risk of POCD at 1 year after surgery.

However, patients with moderate-to-high risk of OSA had a slightly higher incidence of POD (OR, 1.26; 95% CI, 0.29–5.40; *p* = 0.759), POCD at discharge (OR, 1.74; 95% CI, 0.56–5.45; *p* = 0.342), 1 month (OR, 1.29; 95% CI, 0.52–3.19; *p* = 0.577), and 1 year (OR, 1.42; 95% CI, 0.36–5.66; *p* = 0.621) after surgery compared with patients with low risk of OSA, which were not statistically significant differences.

### Moderate-to-high risk of OSA combined with subjective EDS or objective napping was associated with PND

**Figure 3:**
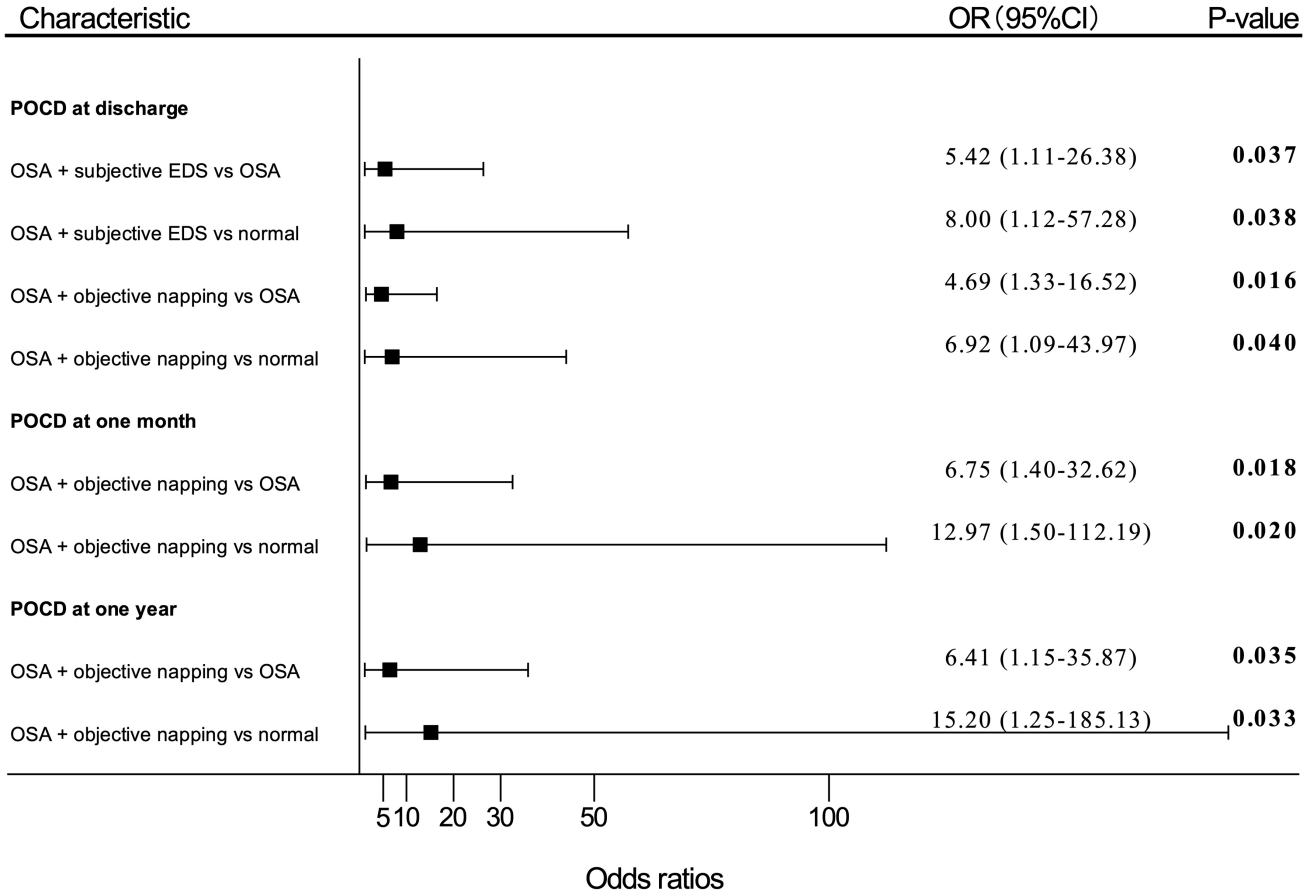
Adjusted (OR) and 95%CI of the effect of OSA with EDS on PND. Estimates were obtained by logistic regression, after adjusting for the significant terms: age, BMI, FS score, MNA-SF score, and MMSE score. “OSA” means patients with moderate-to-high risk of OSA; “normal” means patients with no OSA, no subjective EDS, and no objective napping. OR, odds ratio; OSA, obstructive sleep apnea; EDS, excessive daytime sleepiness; PND, perioperative neurocognitive disorders; BMI, body mass index; FS, frail scale; MNA-SF, mini nutritional assessment-short form; MMSE, Mini-Mental State Examination; POD, postoperative delirium; POCD, postoperative cognitive dysfunction.

The results of the univariate analysis are shown in Supplementary Table S2. Multivariate analysis results in model 1 are shown in Figure 3, and multivariate analysis results in model 2 are shown in Supplementary Figure S2.

Patients with moderate-to-high risk of OSA and subjective EDS showed an OR of 5.42 (95% CI, 1.11–26.38; *p* = 0.037) for POCD developing at discharge compared with patients only having a moderate-to-high risk of OSA. Patients with both moderate-to-high risk of OSA and subjective EDS also presented an OR of 8.00 (95% CI, 1.12–57.28; *p* = 0.038) for risk of POCD at discharge compared with patients without moderate-to-high risk of OSA, subjective EDS, and objective napping.

Patients with moderate-to-high risk of OSA and objective napping showed an OR of 4.69 (95% CI, 1.33–16.52; *p* = 0.016) for POCD at discharge, an OR of 6.75 (95% CI, 1.40–32.62; *p* = 0.018) for POCD at 1-month, and an OR of 6.41 (95% CI, 1.15–35.87; *p* = 0.035) for POCD at 1-year after surgery compared with patients with moderate-to-high risk of OSA alone, respectively. Also, patients with both moderate-to-high risk of OSA and objective napping presented an OR of 6.92 (95% CI, 1.09–43.97; *p* = 0.040) for POCD at discharge, an OR of 12.97 (95% CI, 1.50–112.19; *p* = 0.020) for POCD at 1-month, and an OR of 15.20 (95% CI, 1.25–185.13; p = 0.033) for POCD at 1-year after surgery compared patients without moderate-to-high risk of OSA, subjective EDS, and objective napping, respectively.

## Discussion

To our knowledge, this is the first prospective cohort study to explore whether the moderate-to-high risk of OSA and moderate-to-high risk of OSA combined with EDS are associated with PND. We found that patients with moderate-to-high risk of OSA had a slightly higher incidence of PND than those with low-risk OSA, which were not statistically significant differences. However, moderate-to-high risk of OSA combined with subjective EDS significantly increased the risk of POCD at discharge. Furthermore, moderate-to-high risk of OSA combined with objective napping increased the risk of POCD at discharge, 1 month, and 1 year after surgery.

Our results did not show a significant association between moderate-to-high risk of OSA and POD, which were consistent with three studies that found no association between high preoperative risk of OSA assessed by the STOP-BANG questionnaire and the incidence of POD (Wang S. et al., 2018; King et al., 2020). However, there were four cohort studies suggested that a higher apnea-hypopnea index will increase the risk of POD development (Flink et al., 2012; Roggenbach et al., 2014; Nadler et al., 2017; Wu et al., 2022). The inconsistency of the results may be due to the limitation of the STOP-BANG questionnaire to distinguish the severity of OSA. Previous studies showed that the STOP-BANG had consistently high levels of sensitivity and low levels of specificity when compared to polysomnography (PSG) regardless of the patient population (Miller et al., 2018). Therefore, some patients with a low risk of OSA were wrongly diagnosed with a moderate-to-high risk of OSA, which caused the difference in POD between OSA and non-OSA groups to be not obvious. Besides, the CAM-S short form used by non-psychiatrists to detect POD will have relatively low sensitivity (Inouye et al., 2014). POD occurring at other times may be overlooked except for the evaluation time. Then, the incidence of POD in the OSA group was 8% in our study, which was much lower than the 53% and 44% reported by previous research (Flink et al., 2012; King et al., 2020). Our study may lack the power to detect this small difference in POD between the two groups to some extent. However, it is also necessary to formally implement preoperative assessment of the STOP-BANG questionnaire into the standard for POD risk stratification regarding patients undergoing surgery who do not undergo routine PSG. Because PSG, which with high expense, relative inaccessibility, and time consumption, can delay the diagnosis and treatment of OSA. Future studies are needed to explore the association between OSA and POD and clarify the relevance of preoperative OSA and the accuracy of the STOP-BANG questionnaire when used for POD risk stratification.

Our study firstly assessed the effect of moderate-to-high risk of OSA on the risk of POCD at discharge, 1 month, and 1 year after surgery, and the damaging effect of OSA on POCD during 1-year follow-up periods has no statistical significance. However, one clinical trial retrospectively analyzed clinical data and found that OSA diagnosed by PSG may adversely affect postoperative cognitive function before discharge (Wu et al., 2022). In addition to the limitations of the STOP-BANG questionnaire to identify OSA risk, the insensitivity of questionnaires we used to detect mild cognitive impairment may cause difficulty in recognizing the slight differences between the two groups. Recommendations for the measurement of POCD are neuropsychological tests, usually in the form of multiple tests administered as a test battery (Evered et al., 2018). Another explanation is that the incidence of POCD depends on the type of surgery and surgical populations. Patients undergoing joint replacement are more prone to PND than patients with gastrointestinal surgery, (Postler et al., 2011; Norkienė et al., 2013; Krenk et al., 2014; Aceto et al., 2015; Goettel et al., 2017; Kotfis et al., 2018; Wang C. G. et al., 2018; Chaiwat et al., 2019; Guo et al., 2020; Kang et al., 2020; Wang et al., 2020; Yang et al., 2020; Li et al., 2021) and our study may need more sample size to detect differences in PND between the two groups. Furthermore, previous mouse models also confirmed that chronic intermittent hypoxia could worsen cognitive performance during the first 4 days after surgery (Dong et al., 2018; Zhang et al., 2019; Mei et al., 2020). The advantage of animal models is that they permit a consistent, often severe, level of risk exposure and complete mitigation. Therefore, the presence of OSA may play an important role in POCD in clinical practice, and the relationship between OSA and POCD within 1 year after surgery warrants future exploration in a prospective cohort study with a large sample size and the gold standard for OSA and POCD should be used.

Our study found that moderate-to-high risk of OSA with subjective EDS was associated with POCD at discharge, and moderate-to-high risk of OSA with objective napping was a predictor for POCD at discharge, 1 month, and 1 year after surgery. The mechanism of why the moderate-to-high risk of OSA with EDS may increase the incidence of PND is unclear. Only three animal experiments found that POCD in rats exposed to chronic intermittent hypoxia might be attributed to neuroinflammation (marked by microglial activation and IL-1β levels) (Dong et al., 2018; Zhang et al., 2019; Mei et al., 2020). Significantly, one study involving 58 OSA patients suggested that OSA with objective EDS was the more severe phenotype of the disorder associated with low-grade inflammation (Li et al., 2017). Another clinical study found that EDS might be a potentially useful clinical marker to identify patients with severe OSA at risk of metabolic syndrome (Huang et al., 2016). A recent study indicated that subjective EDS in OSA may be related to neuronal injury and disruptions in the dopaminergic system (Paik et al., 2014). Neuroimaging studies of brain structure also have resulted in a consensus that white matter, gray matter, and hippocampal damage are present in patients with OSA and EDS (Lal et al., 2021). In summary, OSA with EDS represents a severe phenotype and is identified as an important contributor to poor outcomes. Surgical stress can further aggravate inflammation and metabolic disorder and accelerate neuronal injury/apoptosis, ultimately causing cognitive impairment. Importantly, these findings emphasize the need for clinicians to pay particular attention to OSA in combination with EDS in elderly patients, which may impact the development of PND and clinical decision-making regarding treatment. Furthermore, the results of this study do not support the use of OSA alone to make individual treatment decisions, but the combined use of the STOP-BANG questionnaire and subjective EDS/ longer objective napping to predict PND.

Another important thing to note is that, in our study, moderate-to-high risk of OSA with subjective EDS only predicted POCD at discharge. But the moderate-to-high risk of OSA with objective naps predicted POCD at discharge, 1 month, and 1 year after surgery. The reason might be that subjective EDS only reflect perceived sleepiness but is less likely to capture unplanned naps among older adults, which could lead to underestimation of napping behaviors. One study showed that older adults rarely reported EDS and did not always recognize napping or how much they napped (McPhillips et al., 2020). Some studies have compared the effect of objective daytime naps and subjective EDS on cognitive dysfunction (Bolitho et al., 2013; Gotts et al., 2015). The findings suggested that daytime actigraphy, a non-invasive and inexpensive objective measure of daytime sleep, could predict patients with cognitive dysfunction rather than subjective EDS. In addition, subjective EDS and objective napping may reflect two different central nervous system processes. The ESS captures the subjective complaint of daytime sleepiness resulting from impaired sustained attention (Yun et al., 2015) whereas longer daytime nap is associated with an increased level of inflammation or abnormal brain metabolites (Spira et al., 2018). This hypothesis is supported by several studies indicating that objective, but not subjective, sleepiness is associated with inflammation in patients with OSA (Li et al., 2017; Mehra et al., 2017). Therefore, objective napping, compared to subjective EDS, is a better predictor of daytime impairment and PND risks. There is a need to use daytime naps in the routine evaluation of older adults with OSA before surgery.

This study has several limitations. Firstly, we evaluated OSA using STOP-BANG but not polysomnography, which may underestimate the severity of OSA, especially in the presence of high AHI values. The association between preoperative OSA, especially severe OSA combined with EDS, and PND may be greater than what we observed. Secondly, our study used actigraphy to obtain objective daytime naps, but MSLT is considered the standard gold method for the objective measure of daytime sleepiness. It is unknown if actigraphy has the same effect as MSLT for measuring objective EDS. However, given that MSLT is a cumbersome and expensive measure of EDS, there is a need to validate easy-to-use and inexpensive methods of objective EDS to be used in the routine evaluation of OSA patients. This work provided efference to actigraphy applications in older adults with OSA before surgery. Thirdly, we did not use a neuropsychological test battery, considered the gold standard for PND diagnosis, to assess PND. In addition, we did not use the Montreal Cognitive Assessment (MoCA), which has a higher sensitivity than MMSE among elderly patients, to evaluate cognitive performance at discharge. The limitation of measuring tools could significantly underestimate the number of patients with PND. The association between OSA, EDS, or OSA with EDS and PND may exist undetected. Fourthly, while we controlled for learning effects of MMSE and TICS, patients recovered above baseline levels at 1 year suggesting either that this control was incomplete, or that patients had depressed cognitive levels at baseline likely due to pain, pre-admission narcotics, other psychoactive medications, or immobility. Fifthly, the incidence of POD in our study is 7%, which is lower than the rate of POD reported in previous studies. Our study may lack the power to detect this small difference in POD between the two groups to some extent. Sixthly, many factors, including postoperative pain, anxiety, and discomfort, may mediate the association between sleep and long-term cognitive outcomes. While we wanted to avoid over-controlling for variables that might be intermediaries between sleep and cognitive decline, such control will be important in future work to clarify mechanisms and targets for intervention. Finally, our study has some degree of loss to follow-up despite our efforts to assess all participants. The rate of loss to follow-up at discharge, 1-month and 1-year after surgery were similar in both OSA and non-OSA groups, and there was no difference in baseline data between patients lost to follow-up and those lost to follow-up. Thus, our results are reasonable and believable, assuming nondifferential misclassification.

In conclusion, our study suggested that preoperative moderate-to-high risk of OSA combined with EDS could predict a higher risk of POCD at discharge, 1 month, and 1 year after surgery. However, moderate-to-high risk of OSA alone could not precit the development of PND within 1 year after surgery. Given that our study is the first to examine the role of moderate-to-high risk of OSA and moderate-to-high risk of OSA with EDS on the development of PND, more research is needed to explore the effect of OSA and OSA with EDS on PND in independent prospective cohorts. Furthermore, our results suggest that measures of moderate-to-high risk of OSA and EDS are of clinical utility in the identification of high-risk PND in the elderly. Preventions and treatments against OSA and EDS should be investigated and implied to maintain cognitive functional capacity in elderly patients within 1 year after surgery.

## Data availability statement

The datasets presented in this study can be found in online repositories. The names of the repository/repositories and accession number(s) can be found in the article/Supplementary material.

## Ethics statement

The studies involving human participants were reviewed and approved by the ethical approval from the institutional review board of West China Hospital. The patients/participants provided their written informed consent to participate in this study.

## Author contributions

WW and XH initiated the idea for this article and prepared the final copy of the manuscript. GW is responsible for taking pictures and tables. WW and YW took responsibility for collecting patient data. LP and QC took responsibility for reviewing this article. All authors contributed to the article and approved the submitted version.

## Funding

Funding for this study was provided by grant no. 2021YFS0155 from the Fund program Science and Technology Department Program of Sichuan Province (Chengdu, China), grant no. 2020HXBH020 from the Post-Doctor Research Project of West China Hospital (Chengdu, China), grant no. 2018YFC2001802-1 from Ministry of Science and Technology Project of the People’s Republic of China, grant no. 20PJ022 from the Science and Technology Project of the Health Planning Committee of Sichuan (Chengdu, China), and grant no. 2022YFS0267 from the Fund program Science and Technology Department Program of Sichuan Province (Chengdu, China).

## Conflict of interest

The authors declare that the research was conducted in the absence of any commercial or financial relationships that could be construed as a potential conflict of interest.

## Publisher’s note

All claims expressed in this article are solely those of the authors and do not necessarily represent those of their affiliated organizations, or those of the publisher, the editors and the reviewers. Any product that may be evaluated in this article, or claim that may be made by its manufacturer, is not guaranteed or endorsed by the publisher.
