# Corrigendum: Moderate-to-high risk of obstructive sleep apnea with excessive daytime sleepiness is associated with postoperative neurocognitive disorders: a prospective one-year follow-up cohort study

**DOI:** 10.3389/fnins.2024.1359908

**Published:** 2024-02-13

**Authors:** Wenwen Wu, Lihui Pu, Xiuying Hu, Qian Chen, Guan Wang, Yanyan Wang

**Affiliations:** ^1^Innovation Center of Nursing Research, Nursing Key Laboratory of Sichuan Province, West China School of Nursing, West China Hospital, Sichuan University, Chengdu, Sichuan, China; ^2^Innovation Center of Nursing Research, Nursing Key Laboratory of Sichuan Province, West China Hospital, Sichuan University, Chengdu, Sichuan, China; ^3^Menzies Health Institute Queensland & School of Nursing and Midwifery, Griffith University, Brisbane, QL, Australia; ^4^Department of Geriatrics and National Clinical Research Center for Geriatrics, West China School of Nursing, West China Hospital, Sichuan University, Chengdu, Sichuan, China; ^5^Science and Technology Department, National Clinical Research Center for Geriatrics, West China Hospital, Sichuan University, Chengdu, Sichuan, China

**Keywords:** obstructive sleep apnea, excessive daytime sleepiness, perioperative neurocognitive disorders, older adults, surgery

In the published article, there was an error in affiliation(s) 1. Instead of “West China School of Nursing, Sichuan University, Chengdu, Sichuan, China,” it should be “Innovation Center of Nursing Research, Nursing Key Laboratory of Sichuan Province, West China School of Nursing, West China Hospital, Sichuan University, Chengdu, Sichuan, China”.

The authors apologize for this error and state that this does not change the scientific conclusions of the article in any way. The original article has been updated.

